# Supplementary material for: Women infertility and common mental disorders: A cross-sectional study from North India
Source: PLoS One. 2023 Jan 5;18(1):e0280054. doi: 10.1371/journal.pone.0280054 (PMC9815660; doi:10.1371/journal.pone.0280054)
Supplement: Attachment — Submitted filename: PONE-D-22-16063_reviewer comments.pdf [file pone.0280054.s001.pdf]

PLOS ONE

Women Infertility and common mental disorders: A cross-sectional study from North India.

--Manuscript Draft--

**Manuscript Number:**	PONE-D-22-16063
**Article Type:**	Research Article
**Full Title:**	Women Infertility and common mental disorders: A cross-sectional study from North India.
**Short Title:**	Women Infertility and common mental disorders
**Corresponding Author:**	Chakraverti Mahajan, Ph.D<br>University of Delhi Department of Anthropology<br>New Delhi, Delhi INDIA
**Keywords:**	Infertility, stress, anxiety, depression, women, fertility
**Abstract:**	Background: Infertility is a very distressing condition. It is often associated with long-term stress, which can emerge as anxiety and depression.<br>Aim: To understand the effect of socio-demographic variables, reproductive trajectories, and lifestyle variables on stress, depression, and anxiety independently and to understand the relationship of psychological variables with each other among infertile and fertile women.<br>Methods: This cross-sectional study recruited 500 women which included 250 primary infertile cases and 250 age-matched fertile controls of the age group 22-35 years. A pretested modified interview schedule was administered which included demographic variables, lifestyle variables, and reproductive trajectories. In addition, psychological tools like PSS, GAD-7, and PHQ-9 were used to collect the data pertaining to Stress, anxiety, and depression, respectively. Data analysis was performed with the statistical software version SPSS, IBM version 24.<br>Results: The severity of stress, anxiety, and depression are more among infertile women as compared to fertile women. None of the demographic and lifestyle variables were associated with stress, anxiety, and depression among infertile women. Only reproductive trajectories were found to be causing stress, anxiety, and depression respectively among infertile women. In addition, stress is leading to both anxiety and depression among infertile women but only to depression in fertile women.<br>Conclusion: Infertile women should be counselled by medical experts regarding reproductive trajectories. Infertile couples should be guided and counselled to incorporate mental health screening and treatment in their routine check-up.
**Order of Authors:**	Navjot Kamboj, PhD Scholar
	Kallur Nava Saraswathy, PhD
	Sweta Prasad, PhD Scholar
	Nandita Babu, PhD
	Manju Puri, MBBS, MD
	APOORVA SHARMA
	Sukriti Dhingra, MA Psychology
	Mohinder Pal Sachdeva, PhD
	Chakraverti Mahajan, Ph.D
**Additional Information:**	
**Question**	**Response**
**Financial Disclosure**<br><br>Enter a financial disclosure statement that	The authors received no specific funding for the publication of this work.

describes the sources of funding for the work included in this submission. Review the submission guidelines for detailed requirements. View published research articles from *PLOS ONE* for specific examples.<br><br>This statement is required for submission and **will appear in the published article** if the submission is accepted. Please make sure it is accurate.<br><br>**Unfunded studies**<br>Enter: *The author(s) received no specific funding for this work.*<br><br>**Funded studies**<br>Enter a statement with the following details:<br>• Initials of the authors who received each award<br>• Grant numbers awarded to each author<br>• The full name of each funder<br>• URL of each funder website<br>• Did the sponsors or funders play any role in the study design, data collection and analysis, decision to publish, or preparation of the manuscript?<br>• **NO** - Include this sentence at the end of your statement: *The funders had no role in study design, data collection and analysis, decision to publish, or preparation of the manuscript.*<br>• **YES** - Specify the role(s) played.<br><br>* typeset	
**Competing Interests**<br><br>Use the instructions below to enter a competing interest statement for this submission. On behalf of all authors, disclose any competing interests that could be perceived to bias this work—acknowledging all financial support and any other relevant financial or non-financial competing interests.<br><br>This statement is required for submission and **will appear in the published article** if the submission is accepted. Please make sure it is accurate and that any funding	The authors have declared that no competing interests exist.

sources listed in your Funding Information later in the submission form are also declared in your Financial Disclosure statement.<br><br>View published research articles from *PLOS ONE* for specific examples.<br><br>**NO authors have competing interests**<br><br>Enter: *The authors have declared that no competing interests exist.*<br><br>**Authors with competing interests**<br><br>Enter competing interest details beginning with this statement:<br><br>*I have read the journal's policy and the authors of this manuscript have the following competing interests: [insert competing interests here]*<br><br>* typeset	
**Ethics Statement**<br><br>Enter an ethics statement for this submission. This statement is required if the study involved:<br><br>• Human participants<br>• Human specimens or tissue<br>• Vertebrate animals or cephalopods<br>• Vertebrate embryos or tissues<br>• Field research<br><br>Write "N/A" if the submission does not require an ethics statement.<br><br>General guidance is provided below. Consult the submission guidelines for detailed instructions. **Make sure that all information entered here is included in the Methods section of the manuscript.**	The Ethic Committee of the Lady Hardinge Medical College, Delhi and the Department of Anthropology, University of Delhi, Delhi approved the present study.

Powered by Editorial Manager® and ProduXion Manager® from Aries Systems Corporation

**Format for specific study types**<br><br>**Human Subject Research (involving human participants and/or tissue)**<br>• Give the name of the institutional review board or ethics committee that approved the study<br>• Include the approval number and/or a statement indicating approval of this research<br>• Indicate the form of consent obtained (written/oral) or the reason that consent was not obtained (e.g. the data were analyzed anonymously)<br><br>**Animal Research (involving vertebrate animals, embryos or tissues)**<br>• Provide the name of the Institutional Animal Care and Use Committee (IACUC) or other relevant ethics board that reviewed the study protocol, and indicate whether they approved this research or granted a formal waiver of ethical approval<br>• Include an approval number if one was obtained<br>• If the study involved *non-human primates*, add *additional details* about animal welfare and steps taken to ameliorate suffering<br>• If anesthesia, euthanasia, or any kind of animal sacrifice is part of the study, include briefly which substances and/or methods were applied<br><br>**Field Research**<br><br>Include the following details if this study involves the collection of plant, animal, or other materials from a natural setting:<br>• Field permit number<br>• Name of the institution or relevant body that granted permission	
**Data Availability**<br><br>Authors are required to make all data underlying the findings described fully available, without restriction, and from the time of publication. PLOS allows rare exceptions to address legal and ethical concerns. See the PLOS Data Policy and FAQ for detailed information.	No - some restrictions will apply

Powered by Editorial Manager® and ProduXion Manager® from Aries Systems Corporation

A Data Availability Statement describing where the data can be found is required at submission. Your answers to this question constitute the Data Availability Statement and **will be published in the article**, if accepted.<br><br>**Important:** Stating 'data available on request from the author' is not sufficient. If your data are only available upon request, select 'No' for the first question and explain your exceptional situation in the text box.<br><br>Do the authors confirm that all data underlying the findings described in their manuscript are fully available without restriction?	
**Describe where the data may be found in full sentences. If you are copying our sample text, replace any instances of XXX with the appropriate details.**<br><br>• If the data are **held or will be held in a public repository**, include URLs, accession numbers or DOIs. If this information will only be available after acceptance, indicate this by ticking the box below. For example: *All XXX files are available from the XXX database (accession number(s) XXX, XXX.).*<br>• If the data are all contained **within the manuscript and/or Supporting Information files**, enter the following: *All relevant data are within the manuscript and its Supporting Information files.*<br>• If neither of these applies but you are able to provide **details of access elsewhere**, with or without limitations, please do so. For example:<br><br>*Data cannot be shared publicly because of [XXX]. Data are available from the XXX Institutional Data Access / Ethics Committee (contact via XXX) for researchers who meet the criteria for access to confidential data.*<br><br>*The data underlying the results presented in the study are available from (include the name of the third party*	Data cannot be shared publicly because of ethical issues. Data are available from the Department of Anthropology, University of Delhi Institutional Data Access / Ethics Committee (contact via cmahajan@anthro.du.ac.in) for researchers who meet the criteria for access to confidential data.

Powered by Editorial Manager® and ProduXion Manager® from Aries Systems Corporation

and contact information or URL).<br>• This text is appropriate if the data are owned by a third party and authors do not have permission to share the data.<br><br>* typeset	
Additional data availability information:	

Manuscript Click here to access/download;Manuscript;PLOS ONE Manuscript.docx

Title: Women Infertility and common mental disorders: A cross-sectional study from North India.

Navjot Kamboj[1], Kallur Nava Saraswathy[1], Sweta Prasad[1], Nandita Babu[2], Manju Puri[3] Apoorva Sharma[1], Sukriti Dhingra[4], Mohinder Pal Sachdeva[1], Chakraverti Mahajan[1]*.

1.Department of Anthropology, University of Delhi, India

2.Department of Psychology, University of Delhi, India

3.Department of Obstetrics and Gynaecology, Lady Hardinge Medical College, Delhi, India

4.Rehabilitation Council of India, New Delhi, India.

***Corresponding author:** Phone: 9015062967, E-Mail- cmahajan@anthro.du.ac.in

Abstract:

Background: Infertility is a very distressing condition. It is often associated with long-term stress, which can emerge as anxiety and depression.

Aim: To understand the effect of socio-demographic variables, reproductive trajectories, and lifestyle variables on stress, depression, and anxiety independently and to understand the relationship of psychological variables with each other among infertile and fertile women.

Methods: This cross-sectional study recruited 500 women which included 250 primary infertile cases and 250 age-matched fertile controls of the age group 22-35 years. A pretested modified interview schedule was administered which included demographic variables, lifestyle variables, and reproductive trajectories. In addition, psychological tools like PSS, GAD-7, and PHQ-9 were used to collect the data pertaining to Stress, anxiety, and depression, respectively. Data analysis was performed with the statistical software version SPSS, IBM version 24.

Results: The severity of stress, anxiety, and depression are more among infertile women as compared to fertile women. None of the demographic and lifestyle variables were associated with stress, anxiety, and depression among infertile women. Only reproductive trajectories

were found to be causing stress, anxiety, and depression respectively among infertile women. In addition, stress is leading to both anxiety and depression among infertile women but only to depression in fertile women.

Conclusion: Infertile women should be counselled by medical experts regarding reproductive trajectories. Infertile couples should be guided and counselled to incorporate mental health screening and treatment in their routine check-up.

Keywords: Infertility, stress, anxiety, depression, women, fertility

Background:

According to the World Health Organisation "Infertility is a condition of the male or female reproductive system described by the inability to conceive after 12 months or more of unprotected sexual activity" [1]. Infertility is a chronic struggle for couples and it affects about 10 to 12% of couples worldwide [2]. Infertility in women can be caused by many different factors such as a damaged or blocked fallopian tube, endometriosis, PCOS, and hormonal imbalance [3]. It may work as a painful emotional experience and cause a lot of psychological issues or mental disorders such as stress, anxiety, depression, diminished self-esteem, and reduced quality of life [4]. Studies revealed that most infertile women were found to have higher levels of stress, anxiety, and depression [5,6], and these disorders were found to be more associated with infertility as compared to other mental disorders [7].

Stress is a physical and emotional reaction that people have when they are confronted with life upheavals. Long time stress can cause a variety of health concerns including depression, anxiety, and other mental illnesses[8]. The association between stress and infertility has been debated for years [4]. Stress can affect fertility in women by suppressing luteinizing hormone, increasing serum cortisol levels preventing implantation of a fertilized egg, and reducing egg quality [9]. If the stress is continued and prolonged it can result in anxiety and depression [6].

Anxiety and depressive disorders are bidirectional risk factors for one another [10]. Various studies concluded that anxiety leads to depression and thus it is a unidirectional phenomenon [11-13], whereas others reported that depression also led to later anxiety suggesting its bidirectional mechanism [14,15].

A Study by Magalhaes et al. (2010) has revealed that the corticotropin-releasing factor receptor 1(CRFR1) and serotonin receptor (5-HTR) are related to anxiety and depression respectively. CRFR1 works to increase the number of 5-HTRs on the cell surface in the brain, which can cause abnormal brain signaling [16]. Since CRFR1 activation leads to anxiety in response to stress and 5-HTRs leads to depression. hence, one can assume that biological stress leads to anxiety which in turn leads to depression.

The relationship between psychological factors (stress, anxiety, and depression) and infertility is rather complex, multifactorial, and bidirectional [17]. Thus, the present study aims to capture the prevalence of stress, depression, and anxiety among infertile and fertile women and to understand the relationship of psychological variables with each other (i.e. Stress and depression and stress and anxiety) among infertile and fertile women. Attempt was also made to understand the effect of socio-demographic variables, reproductive trajectories, and lifestyle variables on stress, depression, and anxiety.

Methods:

The present study is a part of a Project titled "Infertility and its psychological impact: A case-control study from North India" funded by the National Commission for Women of India, Government of India. Data was collected from 500 females, including 250 infertile and 250 age-matched fertile married women with the age group of 22-35 years from August 2020 to February 2021 after obtaining the ethical clearance from the Department of Anthropology and Lady Hardinge Medical college & its associated Hospital, New Delhi. After taking a written

informed consent the data of infertile women was collected from the Gynaecology Outpatient Department (OPD) of Lady Hardinge Medical College and Smt. Suchita Kriplani Hospital, New Delhi, and data of fertile women was collected using a household survey in Delhi. Both Infertile and fertile women were recruited based on strict inclusion and exclusion criteria. The inclusion criteria for infertile women were women seeking infertility treatment and belonging to North India. The inclusion criteria for fertile women were women of the same reproductive age, with successful pregnancy outcomes having children older than one year and also belonging to North India.

A pretested modified interview schedule was used to collect data pertaining to demographic variables (age, educational status, occupational status, social-economic status [18] and family structure), lifestyle variables (sleep pattern, physical activity, exercise), and reproductive trajectories (age at menarche, age at marriage, bleeding during menstruation duration of infertility, adoption of birth control pills, intercourse during fertile period, possible causes of infertility). The psychological tools like Perceived Stress Scale (PSS), Generalized Anxiety Disorder (GAD-7) and Patient Health Questionnaire (PHQ-9) were used to collect the data pertaining to stress, anxiety, and depression, respectively.

Statistical Analysis:

In the present study, as the distribution of data was found to be skewed, so median scores of all psychological variables were considered and Mann- Whitney test was performed to compare the groups. Further, Pearson's chi-square test was used to compare the distribution of stress, anxiety, and depression among infertile and fertile women.

In order to identify the causes of stress, anxiety, and depression among infertile and fertile women linear regression analysis was done. Data analysis was performed with the statistical software SPSS, IBM version 24, and the significance level for all the data was <0.05.

Results:

Median scores of stress, Depression, and Anxiety scores of Infertile and Fertile women.

The median scores compared between the fertile and infertile women revealed that the median levels of stress and anxiety were significantly higher among infertile women as compared to that of fertile women ($p<0.05$). However, the median levels of depression among the fertile and infertile women were found to be similar ($p >0.05$). (Table-1)

Table-1 Median scores of Stress, Depression and Anxiety among Infertile and Fertile women:

Psychological Variables	Infertile Median (IQR)	Fertile Median (IQR)	Mann Whitney Test P-value
Stress (PSS)	21(16-26)	17(12-21)	0.001
Anxiety (GAD-7)	8(5-12)	4 (3-9)	0.001
Depression (PHQ-9)	9(5-13)	8 (5-12)	0.49

IQR- Interquartile range, Level of significance $p<0.05$

Stress, Anxiety, and Depression among infertile and fertile women

The infertile and fertile women were compared in terms of stress, anxiety, and depression with reference to distribution of frequency, which revealed that the individuals with high stress, anxiety, and depression were found to be significantly higher among the infertile women as compared to fertile women ($p<0.05$). (Table-2)

Table 2: Distribution of Stress, Anxiety, and Depression among infertile and fertile women

Psychological Variables	Levels (Scores)	Infertile (250) N (%)	Fertile (250) N (%)	(P- Value)
Stress	Low (0-13)	33(13.2)	88(35.2)	0.001
	Medium (14-26)	160(64.0)	152(60.8)	
	High (27-40)	57(22.8)	10(4.0)	
Anxiety	Mild (0-4)	59(23.6)	189(75.6)	0.001
	Moderate (5-9)	82(32.8)	52(20.8)	
	Moderately Severe (10-15)	69(27.6)	6(2.4)	
	Severe(15-21)	40(16.0)	3(1.2)	
Depression	Normal(0-4)	58(23.2)	191(76.4)	0.001
	Mild (5-9)	82(32.8)	29(11.6)	
	Moderate(10-14)	65(26.0)	23(9.2)	
	Moderately severe(15-19)	38(15.2)	7(2.8)	
	Severe (20-27)	7(2.8)	0(0)	

N is the sample size, Level of significance p<0.05

Distribution of Demographic variables, Lifestyle variables, and Reproductive trajectories among infertile and fertile women on the variables of Stress, Depression, and Anxiety

Further, the difference between non-stressed (low stress) and stressed (medium and high stress), anxious (moderately severe and severe anxiety) and non-anxious (mild and moderate anxiety), and depressed (mild, moderate, moderately severe, and severe depression) and non-depressed (normal) women were compared with respect to demographic, lifestyle variables, and reproductive trajectories, independently in both infertile and fertile women. The duration of infertility and possible causes of infertility in low versus highly stressed, anxious, and depressed infertile women were also compared (Tables 3, 4, and 5).

Demographic variables, Lifestyle variables, and Reproductive trajectories with reference to Stress

The results indicate that infertile women whose husbands were having private jobs or were daily wage earners were found to be more stressed as compared to those who were unemployed or were either having government jobs or their own business ($p=0.001$). The infertile women who rarely exercised were observed to be more stressed as compared to those who exercised frequently ($p=0.006$). The women who had infrequent intercourse during the fertile period were found to be more stressed in comparison to those who had frequent intercourse during the fertile period among the infertile as well as fertile women category ($p=0.01$). In the case of fertile women, those who were extremely active were found to be more stressed as compared to those who were less active or not active at all ($p= 0.003$).

Further, many other variables were similarly distributed among stressed and non-stressed women in both infertile and fertile categories. Among the infertile women, the women in the age group 31-35, those who were either illiterate or were having primary education, those living in a nuclear family, those belonging to upper lower socioeconomic status, those who were not active or were slightly active, those having 6-7 hours of sleep daily, those whose age at menarche was 9-12 years, those having disturbed menstruation, those who got married after 25 years of age, those experiencing heavy bleeding during menstruation, those whose duration of infertility was more than 5 years, those who had never taken birth control pills in the past and those whose possible cause for infertility was cyst were more stressed as compared to other subgroups of the respective variables ($p>0.05$).

Similarly, among the fertile women as well, the women above the age of 30 years, those who were illiterate, those living in the nuclear family, whose husbands having a business, those belonging to lower-middle socioeconomic status, those who sometimes exercised, those having less than 6 hours of sleep daily, those whose age at menarche was 13-16 years, those having

disturbed menstruation, those who got married after 25 years of age, those having normal or less bleeding during menstruation and those who never took birth control pills in the past were more stressed in comparison to other subgroups of the respective variables. However, the observed difference among these variables between stressed and non-stressed women was not found to be statistically significant (p> 0.05). (Table 3)

Table 3: Distribution of Demographic, Lifestyle variables and reproductive trajectories in infertile and fertile women in Stress category

CHARACTERISTICS		INFERTILE WOMEN		P-value	FERTILE WOMEN		P-value
		**Stressed (217) N(%)**	**Non-stressed (33) N(%)**		**Stressed (162) N(%)**	**Non-stressed (88) N(%)**	
**Age (years)**	**Upto 25**	84 (38.7)	16 (48.48)	0.52	63 (38.89)	36 (40.9)	0.87
	**26-30**	95 (43.78)	13 (39.39)		71 (43.83)	39 (44.32)	
	**31-35**	38 (17.52)	4 (12.12)		28 (17.28)	13 (14.78)	
**Educational status**	**illiterate/informal**	27(12.44)	1 (3.03)	0.28	58 (35.8)	23 (26.14)	0.6
	**Primary**	22 (10.14)	1 (3.03)		63 (38.89)	38 (43.18)	
	**Middle**	14 (6.45)	2 (6.06)		27 (16.67)	19 (21.59)	
	**secondary/senior secondary**	41 (18.89)	7 (21.21)		6 (3.70)	3 (3.41)	
	**Higher**	113(52.08)	22 (66.66)		8 (4.94)	5 (5.68)	
**Family Structure**	**Nuclear**	96 (44.24)	14 (42.42)	0.85	126 (77.78)	59 (67.05)	0.06
	**Joint**	121 (55.76)	19 (57.58)		36 (22.22)	29 (32.95)	
**Occupational status**	**Not working**	3 (1.38)	2 (6.06)	**0.001**	3 (1.85)	2 (2.27)	0.08
	**daily wage earner**	68 (31.34)	2 (6.06)		105 (64.81)	57 (64.77)	
	**private job**	88 (40.55)	11 (33.33)		31 (19.13)	25 (28.41)	
	**govt job**	25 (11.52)	9 (27.27)		15 (9.28)	4 (4.55)	
	**Business**	33 (15.21)	9 (27.27)		8 (4.94)	0 (0)	
**Family Income**	**Upper lower**	44 (20.29)	3 (9.09)	0.4	50 (30.86)	30 (34.09)	0.85
	**Lower middle**	49 (22.59)	8 (24.24)		28 (17.29)	13 (14.78)	

	**Upper middle**	124 (57.3)	22 (66.66)		84 (51.9)	45 (51.1)	
**Physical Activity**	**Extremely active**	34 (15.67)	10 (30.30)		68 (41.97)	22 (25.00)	
	**Moderately active**	80 (36.86)	11 (33.33)	0.24	32 (19.75)	20 (22.73)	
	**Active**	55 (25.35)	8 (24.24)		52 (32.1)	43 (48.86)	**0.003**
	**Slightly active**	39 (17.97)	4 (12.12)		10 (6.18)	1 (1.14)	
	**Not active at all**	9 (4.15)	0 (0)		0 (0)	2 (2.27)	
**Exercise**	**Never**	100 (46.08)	19 (57.58)	**0.006**	59 (36.42)	36 (40.91)	
	**Seldom**	29 (13.36)	4 (12.12)		72 (44.44)	42 (47.73)	0.53
	**Sometimes**	60 (27.65)	1 (3.03)		29 (17.91)	10 (11.36)	
	**Often**	9 (4.15)	1 (3.03)		2 (1.23)	0 (0)	
	**Very often**	19 (8.76)	8 (24.24)		0 (0)	0 (0)	
**Sleep pattern(hours)**	**<6**	31 (14.29)	6 (18.18)		24 (14.81)	8 (9.09)	
	**6-7**	86 (39.63)	9 (27.27)	0.42	70 (43.21)	38 (43.19)	0.54
	**7-8**	73 (33.64)	15 (45.45)		65 (40.12)	41 (46.59)	
	**>8**	27 (12.44)	3 (9.09)		3 (1.86)	1 (1.14)	
**Age of menarche(years)**	**9-12**	55 (25.36)	6 (18.18)		43 (26.54)	29 (32.96)	
	**13-16**	154 (70.95)	27 (81.82)	0.31	118 (72.84)	58 (65.9)	0.52
	**Above 16**	8 (3.69)	0 (0)		1 (0.62)	1 (1.14)	
**Disturbed menstruation**	**Yes**	85 (39.17)	12 (36.36)	0.76	5 (3.09)	0 (0)	0.09
	**No**	132 (60.83)	21 (63.64)		157 (96.91)	88 (100)	
**Age of marriage(years)**	**10-17**	21 (9.68)	1 (3.03)		9 (5.55)	7 (7.96)	
	**18-25**	149 (68.67)	28 (84.85)	0.24	145 (89.51)	80 (90.09)	0.42
	**26-32**	46 (21.19)	4 (12.12)		8 (4.94)	1 (1.14)	
	**Above 32**	1 (0.46)	0 (0)		0 (0)	0 (0)	
**Bleeding during menstruation**	**Heavy bleeding**	36 (16.59)	5 (15.15)		56 (34.57)	39 (44.32)	
	**Normal bleeding**	149 (68.66)	23 (69.70)	0.97	85 (52.47)	40 (45.45)	0.31
	**Less bleeding**	32 (14.75)	5 (15.15)		21 (12.96)	9 (10.23)	
**Duration of infertility(years)**	**1-5**	152 (70.04)	30 (90.91)		-	-	
	**6-10**	46 (21.20)	3 (9.09)	0.08	-	-	
	**11-15**	16 (7.37)	0 (0)		-	-	
	**Above 15**	3 (1.39)	0 (0)		-	-	
**Adoption of birth control without consulting doctors**	**Yes**	16 (7.37)	4 (12.12)	0.34	35 (21.6)	20 (22.73)	
	**No**	201 (92.63)	29 (87.88)		127 (78.4)	68 (77.27)	0.84
**Intercourse during fertile period**	**Always**	85 (39.17)	21 (63.64)	**0.01**	1 (0.62)	4 (4.54)	
	**Sometimes**	98 (45.16)	6 (18.18)		88 (54.32)	54 (61.37)	**0.04**
	**Never**	34 (15.67)	6 (18.18)		73 (45.06)	30 (34.09)	
	**Blocked tubes**	29 (13.3)	11 (33.34)	0.36	-	-	

**Possible Causes of infertility**	**Irregular menstruation**	39 (17.90)	5 (15.15)		-	-	
	**PCOS/PCOD**	11 (5.06)	1 (3.03)		-	-	
	**Cyst**	28 (12.90)	2 (6.06)		-	-	
	**Endometriosis, Mass development uterus**	11 (5.08)	1 (3.03)		-	-	
	**Fibroid**	10 (4.60)	2 (6.06)		-	-	
	**Hormonal imbalance**	4 (1.86)	0 (0)		-	-	
	**No menstruation**	9 (4.14)	1 (3.03)		-	-	
	**Thyroid/AIDS / TB**	28 (12.90)	4 (12.12)		-	-	
	**Other reasons***	48 (22.14)	6 (18.18)		-	-	

N=number of individuals, Level of significance $p < 0.05$.

* Uterine infection, Salpingectomy and Tubal surgery

Demographic variables, Lifestyle variables, and Reproductive trajectories with reference to Anxiety

Among the infertile women, those who were illiterate or were having primary education were found to be more anxious as compared to those who had higher education (p=0.03). The infertile women who were slightly active or not active were more anxious as compared to those who were extremely or moderately active (p=0.003). The infertile women who got married before the age of 18 years were found to be more anxious as compared to those who got married after 18 years of age (p=0.02). The women who were infertile for more than 5 years were found to be more anxious in comparison to those who were infertile for less than 5 years (p=0.02). Among the fertile women, those women who were below 25 years of age were found to be more anxious in comparison to those who were older than 25 years (p=0.007). The women who were having disturbed menstruation were found to be more anxious as compared to those having regular menses (p=0.000). The fertile women who were having heavy bleeding during

menstruation were found to be more anxious compared to those who were either having less bleeding or were having normal bleeding during menstruation (p=0.04).

Further, many other variables were similarly distributed among anxious and non-anxious women in both infertile and fertile categories, Among the infertile women, those who were above 30 years of age, those living in a nuclear family, those who were daily wage earners, those belonging to upper lower socioeconomic status, those who seldom exercised, those who were having less than 6 hours of sleep daily, those having age at menarche above 12 years, those having disturbed menses, those experiencing heavy bleeding during menstruation, those who took birth control pills in the past, those having infrequent intercourse during the fertile period and those having irregular menses or cyst as the possible cause of infertility were found to be more anxious as compared to other subgroups of the respective variables. However, the observed difference among these variables between anxious and non-anxious women was not found to be statistically significant (p> 0.05).

Similarly, among the fertile women as well, those women who were illiterate, women living in the nuclear family, whose husbands were daily wage earners, those who were slightly active, those who seldom exercised, those having 7-8 hours of sleep daily, those whose age at menarche was before 12 years of age, those who got married early i.e., before 18 years of age and those who took birth control pills in the past were observed to be more anxious in comparison to the other subgroups of the respective variables. However, the observed difference among these variables between anxious and non-anxious women were not found to be statistically significant (p> 0.05). (Table- 4)

Table 4: Distribution of Demographic, Lifestyle variables and reproductive trajectories in infertile and fertile women in the Anxiety category

CHARACTERISTICS		INFERTILE WOMEN		P-value	FERTILE WOMEN		p-value
		**Anxious (109) N(%)**	**Non-anxious (141) N(%)**		**Anxious (9) N(%)**	**Non-anxious (241) N(%)**	
**Age(years)**	**Upto 25**	45 (41.28)	55 (39.01)	0.70	8 (88.89)	91 (37.76)	**0.007**
	**26-30**	44 (40.37)	64 (45.39)		0 (0)	110 (45.64)	
	**31-35**	20 (18.35)	22 (15.60)		1 (11.11)	40 (16.60)	
**Educational status**	**illiterate/informal**	15 (13.76)	13 (9.22)	**0.03**	5 (55.55)	76 (31.54)	0.6
	**Primary**	16 (14.68)	7 (4.96)		3 (33.33)	98 (40.66)	
	**Middle**	8 (7.34)	8 (5.68)		1 (11.11)	45 (18.67)	
	**secondary/senior secondary**	21 (19.27)	27 (19.15)		0 (0)	9 (3.73)	
	**Higher**	49 (44.95)	86 (60.99)		0 (0)	13 (5.59)	
**Family Structure**	**Nuclear**	48 (44.04)	62 (43.97)	1	8 (88.89)	177 (73.44)	0.3
	**Joint**	61 (55.96)	79 (56.03)		1 (11.11)	64 (26.56)	
**Occupational status**	**Not working**	2 (1.83)	3 (2.13)	0.90	0 (0)	5 (2.07)	0.63
	**daily wage earner**	34 (31.19)	36 (25.53)		8 (88.89)	154 (63.9)	
	**private job**	42 (38.53)	57 (40.43)		1 (11.11)	55 (22.82)	
	**Govt. job**	14 (12.84)	20 (14.18)		0 (0)	19 (7.88)	
	**Business**	17 (15.60)	25 (17.73)		0 (0)	8 (3.32)	
**Family Income**	**Upper lower**	26 (23.86)	21 (14.89)	0.19	3 (33.33)	77 (31.95)	0.86
	**Lower middle**	24 (22.03)	33 (23.40)		2 (22.22)	39 (16.18)	
	**Upper middle**	59 (54.2)	87(61.7)		4(44.44)	125 (51.8)	
**Physical activity**	**Extremely active**	18 (16.51)	26 (18.44)	**0.003**	4 (44.44)	86 (35.68)	0.09
	**Moderately active**	33 (30.26)	58 (41.13)		1 (11.11)	51 (21.16)	
	**Active**	23 (21.10)	40 (28.37)		2 (22.22)	93 (38.59)	
	**Slightly active**	28 (25.67)	15 (10.64)		2 (22.22)	9 (3.73)	
	**Not active at all**	7 (6.4)	2 (1.42)		0 (0)	2 (0.83)	
**Exercise**	**Never**	48 (44.04)	71 (50.35)	0.08	3 (33.33)	92 (38.17)	0.65
	**Seldom**	20 (18.35)	13 (9.22)		6 (66.67)	108 (44.81)	
	**Sometimes**	21 (19.27)	40 (28.37)		0 (0)	39 (16.18)	
	**Often**	6 (5.5)	4 (2.84)		0 (0)	2 (0.83)	
	**Very often**	14 (12.84)	13 (9.22)		0 (0)	0 (0)	
**Sleep pattern(hours)**	**<6**	20 (18.35)	17 (12.06)	0.22	1 (11.11)	31 (12.86)	0.86
	**6-7**	34 (31.19)	61 (43.26)		3 (33.33)	105 (43.57)	
	**7-8**	41 (37.61)	47 (33.33)		5 (55.56)	101 (41.91)	
	**>8**	14 (12.84)	16 (11.35)		0 (0)	4 (1.66)	
	**9-12**	25 (22.94)	36 (25.53)	0.85	3 (33.33)	69 (28.63)	0.92

**Age of menarche(years)**	**13-16**	80(73.39)	101 (70.92)		6 (6.67)	170 (70.54)	
	**Above 16**	4 (3.67)	4 (2.84)		0 (0)	2 (0.83)	
**Disturbed menstruation**	**Yes**	48 (44.04)	49 (34.75)	0.14	2 (22.22)	3 (1.24)	**0.000**
	**No**	61 (55.96)	92 (65.25)		7 (77.78)	238 (98.76)	
**Age of marriage(years)**	**10-17**	16 (14.68)	6 (4.26)	**0.02**	1 (11.11)	15 (6.22)	0.88
	**18-25**	72 (66.05)	105 (74.46)		8(88.89)	217 (90.04)	
	**26-32**	21 (19.27)	29 (20.57)		0 (0)	9 (3.73)	
	**32 above**	0 (0)	1 (0.71)		0 (0)	0 (0)	
**Bleeding during menstruation**	**Heavy bleeding**	23 (21.01)	18 (12.77)	0.97	7 (77.78)	88 (36.51)	**0.04**
	**Normal bleeding**	68 (62.39)	104 (73.05)		2 (22.22)	123 (51.04)	
	**Less bleeding**	18 (16.51)	19 (13.48)		0 (0)	30 (12.45)	
**Duration of infertility**	**1-5**	70 (64.22)	112 (79.43)	**0.02**	-	-	
	**6-10**	27 (24.77)	22 (15.6)		-	-	
	**11-15**	9 (8.26)	7 (4.96)		-	-	
	**Above 15**	3 (2.75)	0 (0)		-	-	
**Adoption of birth control without consulting doctors**	**Yes**	11 (10.09)	9 (6.38)	0.28	3 (33.33)	52 (21.58)	0.42
	**No**	98 (89.91)	132 (93.62)		6 (66.67)	189 (78.42)	
**Intercourse during fertile period**	**Always**	40 (36.70)	66 (46.81)	0.20	0 (0)	5 (2.07)	0.42
	**Sometimes**	52 (47.70)	52 (36.88)		7 (77.78)	135 (56.02)	
	**Never**	17 (15.60)	23 (16.31)		2 (22.22)	101 (41.91)	
**Possible Causes of infertility**	**Blocked tubes**	14 (12.84)	26 (18.44)	0.17	-	-	
	**Irregular menstruation**	27 (24.77)	17 (12.06)		-	-	
	**PCOS/PCOD**	4 (3.67)	8 (5.67)		-	-	
	**Cyst**	16 (14.68)	14 (9.93)		-	-	
	**Endometriosis, Mass development uterus**	4 (3.67)	8 (5.67)		-	-	
	**Fibroid**	3 (2.75)	9 (6.38)		-	-	
	**Hormonal imbalance**	2 (1.83)	2 (1.42)		-	-	
	**No menstruation**	6 (5.5)	4 (2.84)		-	-	
	**Thyroid/ / AIDS/ TB**	12 (11.01)	20 (14.18)		-	-	
	**Other reasons***	21 (19.28)	33 (23.4)		-	-	

N=number of individuals, Level of significance $p < 0.05$.

* Uterine infection, Salpingectomy and Tubal surgery.

Demographic variables, Lifestyle variables, and Reproductive trajectories with reference to Depression

Among the infertile women, those who never exercised were found to be more depressed as compared to those who sometimes or often exercised (p=0.008). The infertile women who were taking more than 8 hours of sleep were more depressed as compared to those who were taking less than 8 hours of sleep (p=0.009). The women having less bleeding during menstruation were found to be more depressed as compared to those who were having heavy bleeding during menstruation in the infertile category (p=0.00). In the case of fertile women, those who were living in a joint family were more depressed as compared to those living in a nuclear family (p=0.009). The fertile women who sometimes exercised were more depressed as compared to those who never exercised or exercised regularly (p=0.01). Those fertile women who were taking more than 7 hours of sleep daily were more depressed as compared to those taking below 7 hours of sleep (p=0.001). Among the fertile women, those who got married early i.e., before 18 years of age were observed to be more depressed as compared to those who got married after 18 years of age (p=0.005). Lastly, the fertile women who were having normal bleeding during menstruation were found to be more depressed as compared to those having less or heavy bleeding (p=0.03).

Further, many other variables were similarly distributed among depressed and non-depressed women in both infertile and fertile category i.e., among the infertile women, those who were below 25 years of age, those who were illiterate, those who were living in a joint family, those husbands were having private jobs, those belonging to upper lower socioeconomic status, those who were slightly active or were not active at all, women whose age at menarche was 13-16

years, those who had disturbed menstruation, those who got married before 18 years of age, those who took birth control pills in the past and those having irregular menstruation and other reasons as their possible causes of infertility were more depressed as compared to other subgroups of the respective variables (p> 0.05).

Similarly, among the fertile women as well, women above the age of 30 years, those who went to middle school, those who were daily wage earners, those belong to upper-middle socioeconomic status, those who were extremely active, women whose age at menarche was below 12 years of age, those having disturbed menses and those who never took birth control pills in the past were more depressed as compared to other subgroups of the respective variables. However, the observed difference among these variables between depressed and non-depressed women was not found to be statistically significant (p> 0.05). (Table-5)

Table 5: Distribution of Demographic, Lifestyle variables and reproductive trajectories in infertile and fertile women in Depression category

CHARACTERISTICS		INFERTILE WOMEN		P-value	FERTILE WOMEN		P-value
		**Depressed** **192** N(%)	**Normal** **58** N(%)		**Depressed** **59** N(%)	**Normal** **191** N(%)	
**Age(years)**	**Upto 25**	82(42.7)	17(29.3)	0.17	21(35.6)	78(40.8)	0.22
	**25-30**	81(42.2)	29(50)		24(40.7)	86(45)	
	**31-35**	29(15.1)	12(20.7)		14(23.7)	27(14.1)	
**Educational status**	**Illiterate/informal**	24(12.5)	4(6.9)	0.69	17(28.8)	64(33.5)	0.18
	**Primary**	17(8.85)	6(10.34)		19(32.2)	82(42.9)	
	**Middle**	13(6.77)	3(5.17)		15(25.4)	31(16.2)	

	**Secondary/Senior secondary**	38(19.79)	10(17.24)		4(6.8)	5(2.6)	
	**Higher**	100(52.08)	35(60.34)		4(6.8)	9(4.7)	
**Family Structure**	**Nuclear**	83(43.23)	27(46.6)	0.65	36(61)	149(78)	
	**Joint**	109(56.77)	31(53.4)		23(39)	42(22)	**0.009**
**Occupational status**	**Not working**	4(2.08)	0(0)		2(3.4)	3(1.6)	
	**Daily wage earner**	53(27.60)	17(29.3)	0.30	43(72.9)	119(62.3)	0.36
	**Private job**	80(41.7)	18(31)		9(15.3)	47(24.6)	
	**Govt job**	24(12.5)	12(20.7)		3(5.1)	16(8.4)	
	**Business**	31(16.15)	11(19)		2(3.4)	6(3.1)	
**Family Income**	**Upper lower**	39(20.31)	8(13.8)	0.29	16(27.1)	64(33.5)	0.05
	**Lower middle**	40(20.83)	17(29.3)		5(8.5)	36(18.8)	
	**Upper middle**	113(58.8)	33(56.8)		38(64.4)	91(47.6)	
**Physical activity**	**Extremely active**	32(16.7)	12(20.7)		27(45.8)	64(33.5)	0.32
	**Moderately active**	67(34.9)	24(41.4)	0.38	10(16.9)	43(22.5)	
	**Active**	49(25.5)	14(24.1)		18(30.5)	77(40.3)	
	**Slightly active**	35(18.2)	8(13.8)		4(6.8)	7(3.7)	
	**Not active at all**	9(4.7)	0(0)		0(0)	0(0)	
**Exercise**	**Never**	100(52.1)	19(32.8)		18(30.5)	77(40.3)	
	**Seldom**	25(13.0)	8(13.8)	**0.008***	24(40.7)	90(47.1)	**0.01***
	**Sometimes**	37(19.3)	24(41.4)		15(25.4)	24(12.6)	
	**Often**	7(3.6)	3(5.2)		2(3.4)	0(0)	
	**Most often**	23(12)	4(6.9)		0(0)	0(0)	
**Sleep pattern(hours)**	**<6**	31(16.15)	6(10.3)		10(16.9)	22(11.5)	
	**6-7**	66(34.35)	29(50)	**0.009***	14(23.7)	94(49.2)	**0.001***
	**7-8**	68(35.42)	20(34.5)		32(54.2)	74(38.7)	
	**>8**	27(14.06)	3(5.2)		3(5.1)	1(0.5)	
**Age of menarche(years)**	**9-12**	46(24)	15(25.9)		24(40.7)	48(25.1)	0.06
	**13-16**	139(72.4)	40(69)	0.82	35(59.3)	141(73.8)	

	**Above 16**	7(3.7)	3(5.2)		0(0)	2(1.0)	
**Disturbed menstruation**	**Yes**	76(39.6)	21(36.2)	0.64	4(6.8)	1(0.5)	0.003*
	**No**	116(60.4)	37(63.8)		55(93.2)	190(99.5)	
**Age of marriage (years)**	**10-17**	21(10.9)	3(5.2)	0.13	9(15.3)	7(3.7)	**0.005***
	**18-25**	133(69.3)	39(67.2)		46(78)	179(93.7)	
	**25-32**	38(19.8)	15(25.9)		4(6.8)	5(2.6)	
	**32 above**	0(0)	1(1.7)		0(0)	0(0)	
**Adoption of birth control without consulting doctors**	**Yes**	16(8.3)	4(6.9)	0.13	10(16.9)	44(23)	0.35
	**No**	176(91.7)	54(93.1)		48(81.4)	147(77)	
**Bleeding during menstruation**	**Heavy bleeding**	10(5.2)	31(53.4)	**0.00***	21(35.6)	74(38.7)	**0.03***
	**Normal bleeding**	152(79.1)	18(31.0)		36(61.0)	89(46.6)	
	**Less bleeding**	30(15.6)	9(15.5)		2(3.4)	28(14.7)	
**Intercourse during fertile period**	**Always**	83(43.2)	25(43.1)	0.26	2(3.4)	3(1.6)	**0.02**
	**Sometimes**	77(40.1)	28(48.3)		42(71.2)	100(52.4)	
	**Never**	32(16.7)	5(8.6)		15(25.4)	88(46.1)	
**Duration of Infertility(years)**	**1-5**	135(70.3)	47(81.0)	0.50			
	**6-10**	42(21.8)	7(12.0)				
	**11-15**	12(6.25)	4(6.8)				
	**Above 15**	3(1.56)	0(0)				
**Possible Causes of infertility**	**Blocked tubes**	32(16.7)	8(13.8)	0.48			
	**Irregular menstruation**	35(18.2)	9(15.5)				
	**PCOS/PCOD**	8(4.2)	4(6.9)				
	**Cyst**	24(12.5)	6(10.3)				
	**Endometriosis, Mass development uterus**	10(5.2)	2(3.4)				
	**Fibroid**	9(4.7)	3(5.2)				

	**Hormonal imbalance**	4(2.1)	0(0)				
	**No menstruation**	8(4.2)	2(3.4)				
	**Thyroid/ / AIDS/ TB**	19(9.9)	13(22.4)				
	**Other reasons***	43(22.4)	11(19.0)				

N=number of individuals, Level of significance p < 0.05.

* Uterine infection, Salpingectomy and Tubal surgery.

Association of statistically significant demographic variables, lifestyle variables, and reproductive trajectories with the psychological variables

Linear regression was performed to understand the association between various demographic variables, lifestyle variables, and reproductive trajectories which were significantly different between stress and non-stressed, anxious and non-anxious, and depressed and non-depressed fertile and infertile women. The results revealed that the infertile women who had intercourse infrequent (sometimes) during the fertile period had significantly increased stress by 2.14 units(p= 0.01) whereas the stress is significantly increased by 0.49 units among fertile women due to extreme physical activity(p=0.01)

Regarding anxiety, women who are infertile for 6-10 years showed a significant increase in anxiety by 2.10 units (p=0.003). Heavy bleeding during menstruation increased anxiety by 0.60 units among fertile women (p=0.04) whereas, less bleeding during menstruation significantly increased depression by 1.21 units among infertile women (p=0.005). Infrequent (sometimes) intercourse during the fertile period showed a mild decrease in depression among fertile women (p=0.04). (Table-6)

Table 6: Linear regression with respect to statistically significant Demographic, lifestyle, and reproductive trajectories among infertile and fertile women.

	Infertile			Fertile		
Dependent Variables	Significant (Independent variable)	B coefficient (Standard error)	P-value	Significant (Independent variable)	B coefficient (Standard error)	P-value
Stress	Infrequent intercourse during the fertile period	2.14 (0.899)	**0.01**	Less Physical Activity	0.496 (0.195)	**0.01**
Anxiety	Higher Duration of infertility	2.10 (0.708)	**0.003**	Heavy Bleeding during menstruation	0.602 0.296	**0.04**
Depression	Less Bleeding during menstruation	1.21 (0.434)	**0.005**	Infrequent Intercourse during fertile period	-0.877 (0.443)	**0.04**

Association of Stress with Depression and Anxiety among Infertile and Fertile Women

Finally, linear regression was performed to understand the association between stress and other psychological variables, i.e., Depression and Anxiety, which revealed that among the infertile women, a unit change in stress significantly increased depression by 0.14 units (p=0.001) and anxiety by 0.10 units (p=0.02). Whereas, among the fertile women, a unit change in stress significantly increases depression by 0.15 units.(0.001)(Table-7)

Table 7: Association of stress with other psychological variables among Fertile and Infertile women

**Psychological Variables**		**Infertile**		**Fertile**	
**Independent Variable**	**Dependent Variable**	**B coefficient (Standard error)**	**P-value**	**B coefficient (Standard error)**	**P- value**
Stress	Depression	0.144(0.044)	0.001	0.155(0.04)	0.001
	Anxiety	0.104(0.045)	0.023	-0.021(0.051)	0.685

Discussion:

The findings of the present study revealed that the prevalence of stress, anxiety, and depression is more among infertile women as compared to fertile women. The results are in concordance with the findings of previous studies which also reported an increased level of stress, anxiety [6,19,20], and depression associated with infertility [21]. Infertility is a condition where a woman experiences social, biological, and cultural trauma, which might act as external stimuli and in turn are likely to cause stress. Long-term stress is an endophenotype for various psychological dysfunctions including anxiety, depression, and cognitive impairment [22].

Further, the distribution of demographic variables, lifestyle variables, and reproductive trajectories among infertile and fertile women vis-a-vis stress, depression, and anxiety was also investigated. In reference to stress, the infertile women whose husbands were private job employees or were daily wage earners were found to be more stressed. This could be attributed to the work-life imbalance, unsatisfactory job roles, and financial instability in addition to the trauma of infertility.

The results also revealed that the infertile women who never exercised were found to be more stressed and more depressed. As observed by the field investigators of the present study, exercise among infertile women is one of the recommendations of the doctors as a part of their treatment protocol specifically for the reduction of PCOS and other related infertility

issues. Some intervention and prospective studies have also demonstrated that lesser exercise results in higher levels of perceived stress [23, 24]. Further, clinical trials have demonstrated that exercise is an effective method for reducing perceived stress, stress symptoms, and quality of life [23]. Among the fertile group, physically active women were found to be more stressed, which could be due to workload, the pressure of household chores, or other family or children-related issues. In support of this, linear regression revealed that extreme physical activity also causes stress among fertile women.

Further, both fertile and infertile women who had infrequent intercourse(sometimes) during the fertile period were found to be more stressed. Even though the relationship between infrequent intercourse and stress might be bidirectional among both infertile and fertile women, but the reasons could be different. In the case of infertile women, when pregnancy does not happen even after trying multiple times over the years, the infertile couples tend to lose interest in sexual activities and develop stress, as a result of which, their sexual desires tend to reduce. Infertile couples often forget that their sexual relationship is also their natural need [25]. Thus, the stress due to infertility leads to infrequent or reduced sexual intercourse amongst infertile couples. Whereas, in the case of fertile women, stress could be due to high physical activity. Because of extreme physical activity and tiredness, women can get stressed, which further leads to infrequent sexual intercourse among the fertile couple. Moreover, less frequent sexual intercourse among both fertile and infertile women leads to the minimal release of endorphins, which is a natural mood booster, thus leading to higher levels of stress [26]. In the present study, linear regression analysis also revealed that infrequent intercourse during the fertile period increases stress among infertile women.

In reference to anxiety, the infertile women who were illiterate or had primary education were found to be more anxious. Traditionally, illiterate or less educated women belonging to the middle or lower class are typically involved in domestic tasks and are

homemakers, so household chores, economic dependency, pressure to conceive from the husband and in-laws may lead to lower self-esteem and self-confidence in them which might give rise to anxiety. A study by Kaur et al. (2021) also reported that the prevalence of anxiety was found to be higher among the illiterate and homemakers compared to working women in India [27].

The infertile women who were physically inactive or only slightly active were more prone to anxiety. These women might be less active due to the underlying causes of infertility such as pain due to endometriosis, painful menses, ovarian cysts, etc., which in turn might be leading to anxiety among them. Studies have also demonstrated that people having a sedentary life or those who were less physically active were found to be more anxious [28,29].

Also, infertile women who got married at an early age were found to be more anxious. Sezgin & Punamaki (2020) also found that the early age at marriage is associated with worse mental health and poor wellbeing [30]. The women who were married young are deprived of their right to make vital decisions about their sexual health and wellbeing. In addition, owing to their infertility, they have to deal with various consequences such as violence, abuse, etc., which in turn might lead to anxiety among them. Again, the women who were infertile for more than 5 years were found to be more anxious. A study by Mirghafourvand et al (2016) also reported that anxiety was the most common after 4–6 years of infertility and especially severe anxiety could be found in those who had infertility for 7-9 years [31]. In the present study, the linear regression also revealed that the higher duration of infertility (6-10 years) is causing anxiety among infertile women.

Moreover, the fertile women below the age of 25 years were found to be more anxious. A study by Rebeca & Rebecca (2013) revealed that the younger age group of Indian women, i.e., below 25 years of age, were more vulnerable to anxiety and had less ability to deal with

responsibilities, early pregnancy, and pregnancy-related mortality and morbidity [32]. It causes psychological issues like stress and anxiety in younger women as compared to older women [33]. The fertile women who were having undisturbed menstruation but heavy bleeding were found to be more anxious. Anxiety among fertile women could be due to pre-menstrual syndrome (PMS), which happens before every period, which in turn is associated with mood regulation and causes anxiety among women during or before menstruation. The heavy bleeding or menorrhagia in fertile women could be due to fibroid, hormonal imbalance, or ovulatory dysfunction. A few studies have also reported that menorrhagia causes psychological problems especially anxiety in women [34]. In the present study, the relationship between heavy bleeding and anxiety is also found after regression analysis among the fertile women.

In reference to depression, infertile women who used to oversleep (> 8 hours) were more depressed. Oversleeping among infertile women could be due to the depression induced by infertility. Studies have found out that depression may contribute to sleep-related issues and people who have experienced depression can have excessive sleepiness [35]. A major factor that could cause oversleeping in a depressed person is an interruption to a person's circadian rhythm [36,37]. The infertile women who had less bleeding were found to be more depressed, there is a possibility that the infertile women might be attributing the cause of their infertility to less bleeding during menstruation, which, in turn, may lead to depression among them. A lesser amount of bleeding is found to be causing depression among infertile women, as has been revealed in linear regression as well.

In addition, the fertile women belonging to the joint family were found to be more depressed. The joint families do not provide enough scope for women to develop qualities of adventure, self-determination, which adversely affects their individuality, originality, and creativity, and this can further cause depression among fertile women [38]. The fertile women who exercised sometimes were found to be more depressed. Depressed people have a lack of

interest in working out because when a person is suffering from depression, exercise is the last thing they want to do. A study by Kandola et al. (2019) reported that people with low aerobic and muscular fitness levels are almost twice as likely to experience depression.[39] Thus, it can be concluded that the relationship between irregular or no exercise and depression is bidirectional. Also, infertile women who slept for 7-8 hours were found to be more depressed. Even though the women were having 7-8 hours of sleep daily, but higher levels of depression could be due to sleep disruptions.

Moreover, fertile women who were married at an early age were found to be more depressed. Marriage at an early age leads to early conception and more physical and mental burden among fertile women. It leads to greater psychological traumas such as loss of self-confidence. Among girls who were receiving education, early marriage tends to impede their education, resulting in severe depression [30]. Fertile women having infrequent (sometimes) intercourse were found to be more depressed and it could be because of less release of endorphins, which is a natural mood booster (released during intercourse), which in turn might be leading to depression [40]. Also, depression might be leading to infrequent intercourse. The neurotransmitters of the brain that trigger more blood flow to the sex organs don’t function properly in depressed people and this, in turn, leads to loss of desire in sexual activity [41]. Finally, fertile women having normal bleeding during menstruation were found to be more depressed. Depression among women with normal bleeding might be due to premenstrual symptoms (PMS). Severe symptoms of PMS can also cause depression among women [42].

In sum, the results of the present study reveal that it is stress that in turn leads to depression as well as anxiety among infertile women. An earlier study also reported that chronic stress can lead to depression and anxiety in both infertile women and men and the depressive disorder often occurs together with anxiety in patients, the cause for both of these are strongly linked to stress [19]. On the other hand, stress only leads to depression and not

anxiety among fertile women. The findings are in line with previous research which revealed that long-term stress or chronic stress can contribute to a mood disorder and depression among females [43].

Finally, in terms of a biological pathway, it is observed that stress leads to anxiety which in turn leads to depression [16]. However, it was found in the present study that stress is leading to both anxiety and depression among infertile women but only to depression in fertile women. These findings are in contradiction to the biological pathway, and need further investigation. Although the present study highlights the prevalence of various common mental disorders among infertile women independently with respect to demographic variables, lifestyles variables, and reproductive trajectories, more research is warranted in this direction to have a greater conviction in our findings.

Conclusion:

The infertile women attending gynecology OPD for treatment need to be counseled regarding reproductive trajectories mainly in terms of less bleeding during menstruation and infrequent intercourse during the fertile period. There is a need to strengthen the infrastructure to educate and eradicate the myths around infertility by providing necessary guidance and counseling to both husbands and wives and incorporating mental health screening and treatment in the routine care of infertile women in India. We should try to build an ecosystem where women are encouraged to start discussing infertility-related issues and concerns openly without any apprehensions.

Abbreviations:

AIDS: Acquired immune deficiency syndrome.

TB: Tuberculosis.

PCOS/PCOD: Polycystic ovarian syndrome or disease.

References:

1. World Health Organization . International Classification of Diseases. 11th Revision (ICD-11) Geneva: WHO.2018.

2. Maroufizadeh S, Ghaheri A, Almasi-Hashiani A, Mohammadi M, Navid B, Ezabadi Z, et al. The prevalence of anxiety and depression among people with infertility referring to Royan Institute in Tehran, Iran: a cross-sectional questionnaire study. Middle east Fertil Soc J. 2018; 23(2):103–106.

3. Harms, R. Infertility: Causes. http://www.mayoclinic.com/health/infertility/DS00310/DSECTION=causes.

4. Rooney KL, Domar AD. The relationship between stress and infertility.Dialogues in clinical neuroscience. 2018; *20*(1): 41–47.

5. Kiani Z, Simbar M, Hajian S, & Zayeri F. The prevalence of depression symptoms among infertile women: a systematic review and meta-analysis. Fertility research and practice.2021; *7*(1):6.

6. Lakatos E, Szigeti JF, Ujma PP, Sexty R, Balog P. Anxiety and depression among infertile women: a cross-sectional survey from Hungary. BMC Womens Health. 2017; 17(1):48.

7. Greil AL, Slauson-Blevins K, McQuillan J. The experience of infertility: a review of recent literature. Sociol Health Illn. 2010;32(1):140
8. Stress | NCCIH (nih.gov). 2020

9. Domar A D. Conquering infertility: Dr. Alice Domar's mind/body guide to enhancing fertility and coping with infertility.Penguin Books. 2004

10. Nicholas C, Jacobson and Michelle G, Newman. Anxiety and Depression as Bidirectional Risk Factors for One Another: A Meta-Analysis of Longitudinal Studies. American psychological association. 2017; 143(11):1155–120.

11. Merikangas KR, Lieb R, Wittchen HU, Avenevoli S, Family and high-risk studies of social anxiety disorder. 2003; 108: 23-47.

12. Rice F, van den Bree MBM,Thapar A. A population-based

    study of anxiety as a precursor for depression in childhood and adolescence. BMC Psychiatry. 2004; 4: 43–44.

13. Hettema JM, Kuhn JW, Prescott CA, Kendler KS. The impact of generalized anxiety disorder and stressful life events on risk for major depressive episodes. Psychological Medicine. 2006; 36: 789–795

14. Silberg JL, Rutter M, Eaves L. Genetic and environmental influences on the temporal association between earlier anxiety and later depression in girls. Biological Psychiatry. 2001;49: 1040–1049

15. Fichter MM, Quadflieg N, Fischer UC, Kohlboeck G. Twenty-five-year course and outcome in anxiety and depression in the Upper Bavarian Longitudinal Community Study. Acta Psychiatrica scandinavica. 2010; 122: 75–85.

16 Magalhaes A, Holmes K, Dale L. CRF receptor 1 regulates anxiety behavior via sensitization of 5-HT2 receptor signaling.Nat Neurosci. 2010;**13:** 622–629

17 Kleanthi G, Maria G. Alexithymia, Stress and Depression in Infertile Women: a Case Control Study. *Mater Sociomed*. 2021;33(1):70-74. doi:10.5455/msm.2021.33.70-74

18 Saleem SM. Modified Kuppuswamy socioeconomic scale updated for the year 2019. *Indian J Forensic Community Med*. 2019; *6*(1): 1-3.

19.Ramezanzadeh F, Aghssa MM, Abedinia N, Zayeri F, Khanafshar N, Shariat M, et al. A survey of relationship between anxiety, depression and duration of infertility.BMC Womens Health. 2004; 4(1):1–7.

20. Iordachescu DA, Paica CI, Boca AE, Gica C, Panaitescu AM, Peltecu G, Veduța A, Gica N. Anxiety, Difficulties, and Coping of Infertile Women. Healthcare (Basel,Switzerland).2021; *9*(4): 466.

21. Vo TM, Tran QT, Le CV, Do TT, Le TM. Depression and associated factors among infertile women at Tu Du hospital, Vietnam: a cross-sectional study. International journal of women's health. 2019;11:343.

22. Sharpley CF. neurobiological pathways Between chronic stress and Depression: Dysregulated Adaptive Mechanisms?. Clinical Medicine Insights: Psychiatry. 2009 Jan;2:CMPsy-S3658.

23Stults-Kolehmainen MA, Sinha R. The effects of stress on physical activity and exercise. Sports medicine. 2014 Jan;44(1):81-121.

24. Koo KM, Kim CJ. The effect of the type of physical activity on the perceived stress level in people with activity limitations. Journal of exercise rehabilitation. 2018 Jun;14(3):361.

25. Mohammadi MR, Farahani FK. Emotional and psychological problems of infertility and strategies to overcome them. Journal of Reproduction & infertility. 2001 Oct 1;2(4).
26. Endorphins: Effects and how to boost them (medicalnewstoday.com) https://www.medicalnewstoday.com/articles/320839.2018.

27. Kaur A, Das M, Solanki HK, Awasthi S, Hyanki A. Anxiety disorder in homemakers of Kumaon Region of Uttarakhand, India. International Journal of Preventive Medicine. 2021;12.

28. Ratey JJ. Spark: The revolutionary new science of exercise and the brain. Hachette UK; 2008 Jan 1.

29. Anderson BJ, Eckburg PB, Relucio KI. Alterations in the thickness of motor cortical subregions after motor-skill learning and exercise. Learning & memory. 2002 Jan 1;9(1):1-9.

30. Sezgin AU, Punamäki RL. Impacts of early marriage and adolescent pregnancy on mental and somatic health: the role of partner violence. Archives of women's mental health. 2020;23(2):155.

31. Mirghafourvand M, Charandabi SM, Jafarabadi MA, Tavananezhad N, Karkhane M. Predictors of health-related quality of life in Iranian women of reproductive age. Applied Research in Quality of Life. 2016 Sep;11(3):723-37.

32. Rebeca J, Rebeca T. Early female marriage in the developing world. Gender & Development. 2003 Jul 1;11(2):9-19.

33 Ahmed1&2 S, Khan S, Alia M, Noushad S. Psychological impact evaluation of early marriages. International journal of endorsing health science research. 2013;1(2):84-6.

34. Meenakshi MA, Bhuvaneshwari M. Menorrhagia Causes Psychological Problems in Women. International Journal of Scientific and Research Publications. 2013;3(6): 1028.

35. Franzen PL, Buysse DJ. Sleep disturbances and depression: risk relationships for subsequent depression and therapeutic implications. Dialogues in clinical neuroscience. 2008 Dec;10(4):473.

36. Nutt D, Wilson S, Paterson L. Sleep disorders as core symptoms of depression. Dialogues in clinical neuroscience. 2008 Sep;10(3):329.

37. The Link Between Oversleeping and Depression – Cleveland Clinic. Accessed on June 25, 2020.

38. Nighat A. Evaluation of the joint family system as a major cause of depression among married women of Sindh. Interdiscip J Contemp Res Bus. 2013;4(10):113-32.

39. Kandola A, Ashdown-Franks G, Stubbs B, Osborn DPJ, Hayes JF. The association between cardiorespiratory fitness and the incidence of common mental health disorders: A systematic review and meta-analysis. J Affect Disord. 2019. 1 (257) :748-757.

40. Berry J. Endorphins: Effects and How to Increase Levels. Medical News Today. 2018.

41. Bruce F. Sexual problems and Depression.2020.

42. McLeod DR, Hoehn- Saric R, Foster GV, Hipsley PA. The influence of premenstrual syndrome on ratings of anxiety in women with generalized anxiety disorder. Acta Psychiatrica Scandinavica. 1993 Oct;88(4):248-51.

43. Khademi A, Alleyassin A, Aghahosseini M, Ramezanzadeh F, Abhari AA. Pretreatment Beck Depression Inventory score is an important predictor for post-treatment score in infertile patients: a before-after study. BMC psychiatry. 2005 Dec;5(1):1-7.

Declaration:

Ethics Approval and Consent to participate:

Obtained the ethical clearance from the Ethics Committee of the Department of Anthropology, University of Delhi and the Ethics Committee of Lady Hardinge Medical college & its associated Hospital, New Delhi. All methods were carried out in accordance with relevant guidelines and regulations. Informed written consent was obtained from all participants included in the study. Informed consent in the form of thumb impression was taken in the precence of the Legally authorized representatives for illiterate participants.

Consent for Publication:
Not applicable.

Availability of data and materials:

The authors are unable to share raw data because ethical approval was not obtained for data sharing of this nature.

Competing Interest:

The authors stated that there are no conflicts of interest regarding the publication of this article.

Funding:

The project is funded by the National Commission for women, Government of India without publication charges.

Authors Contributions

Planning and designing the study, data analysis and the first draft of the manuscript were prepared by Navjot Kamboj, Kallur Nava Saraswathy, Sweta Prasad and Apoorva Sharma. Sukriti Dhingra participated in the data collection and data entry. Chakraverti Mahajan, Nandita Babu, Manju Puri and M.P Sachdeva were involved in the data interpretation and editing. All authors reviewed and edited subsequent drafts of the manuscript and approved the final version.

Acknowledgement:

I am thankful to the the National Commission for Women (NCW), Government of India for funding the project and to the staff of Lady Hardinge Medical College, New Delhi. We express our gratitude to all the participants who cooperated with the research team. I also thank Jyoti Mishra for the idea of the research and research team Shivani Tyagi & Ankita Chandola for carrying out fieldwork in the times of the pandemic.
