# Supplementary material for: Women infertility and common mental disorders: A cross-sectional study from North India
Source: PLoS One. 2023 Jan 5;18(1):e0280054. doi: 10.1371/journal.pone.0280054 (PMC9815660; doi:10.1371/journal.pone.0280054)
Supplement: Attachment — Submitted filename: Response to reviewers.docx [file pone.0280054.s002.docx]

**Response to Reviewers:**

The authors heartly thank the reviewers for their valuables comments on the manuscript titled “Women Infertility and common mental disorders: A cross-sectional study from North India.” The comments given by authors will surely enhance the manuscript and the authors have tried their best to incorporate the appropriate suggestions.

1. Entire manuscript has been formatted according to the journal guidelines.
2. **Abstract:** As suggested the results section has been rephrased to avoid repetition.
3. **Background:**
i. Page no. 3 first paragraph later has been deleted.
ii. Page no. 3 second last para “( i.e. Stress and depression and stress and anxiety) among infertile and fertile women” has been deleted due to repetition.
4. **Methods:**
i. Page no. 4 1^st^ line Suchita has been changed to “Sucheta”
ii. Page no. 4 1^st^ para “Both Infertile and fertile women were recruited based on strict inclusion and exclusion criteria.” has been deleted.
5. **Results:**
i. Page no. 5 1^st^ heading “scores” is deleted.
ii. Page no. 7 last and 2^nd^ last para has been deleted as suggested by the reviewers.
iii. Page no. 13 paragraphs has been deleted as suggested by the reviewers.
iv. Page no. 19 first para has been deleted as suggested by the reviewers.
v. Page no. 23-25 “units” has been changed to times.

**Reviewers comment: How was this calculated?**

vi. Page 23- 25: This was calculated regression analysis. As, regression analysis depicts the relationship between two variables. It is applied in scenarios where the change in the value of the independent variable causes changes in the value of the dependent variable.
6. **Discussion:**

**Reviewers comment: Comparison with other literature**

i. References has been added wherever possible as suggested by the reviewers.
ii. Reference numbering have been changed
7. **References:**
i. References have been added wherever required.
ii. Formatting and numbering has been changed.
